# Supplementary figures and images for: A cathepsin C-like protease mediates the post-translation modification of Toxoplasma gondii secretory proteins for optimal invasion and egress
Source: mBio. 2023 Jun 16;14(4):e00174-23. doi: 10.1128/mbio.00174-23 (PMC10470614; doi:10.1128/mbio.00174-23)

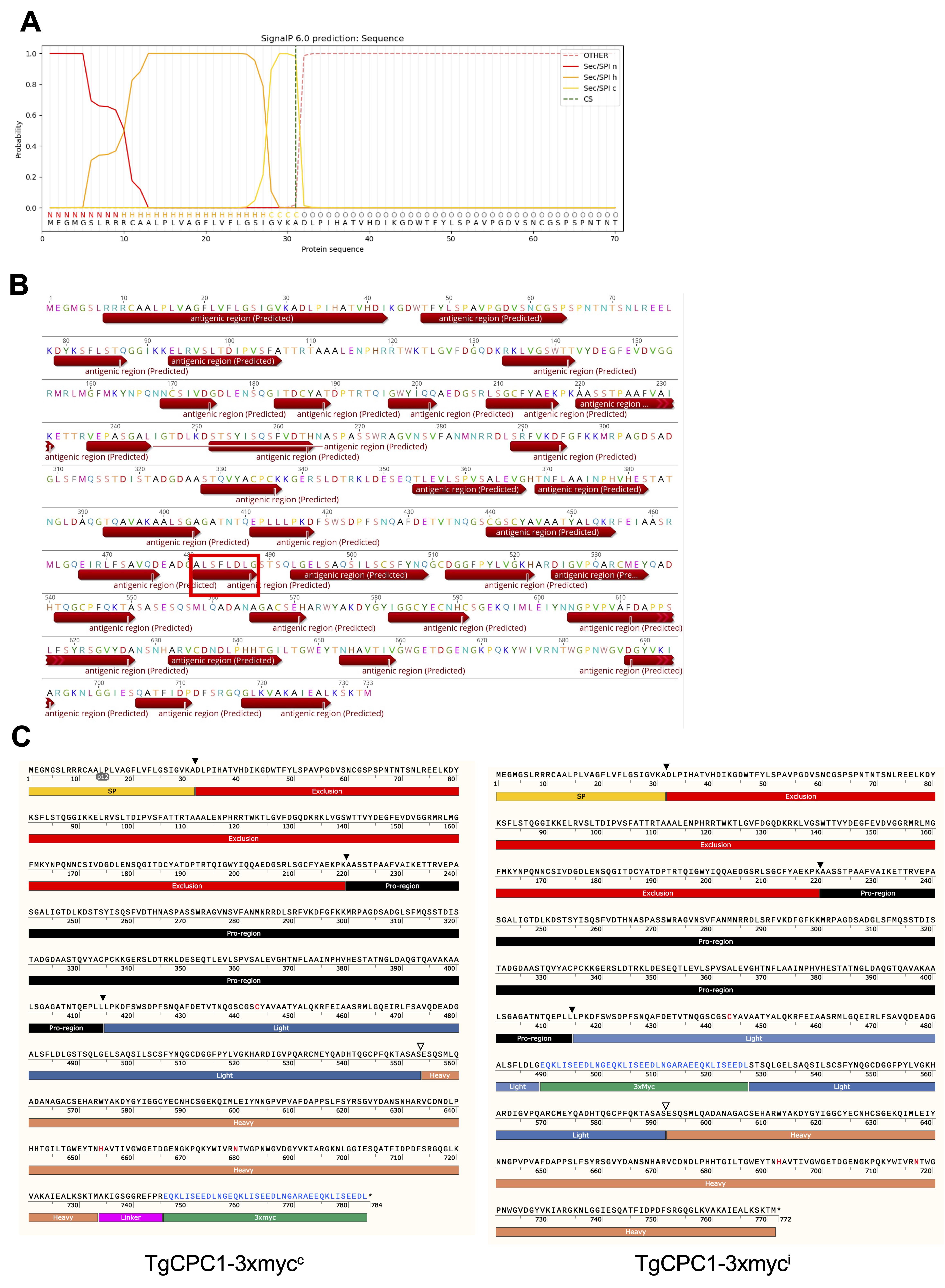

Supplement: Figure S1 — Primary structure and motifs of TgCPC1. [file mbio.00174-23-s0001.tif]

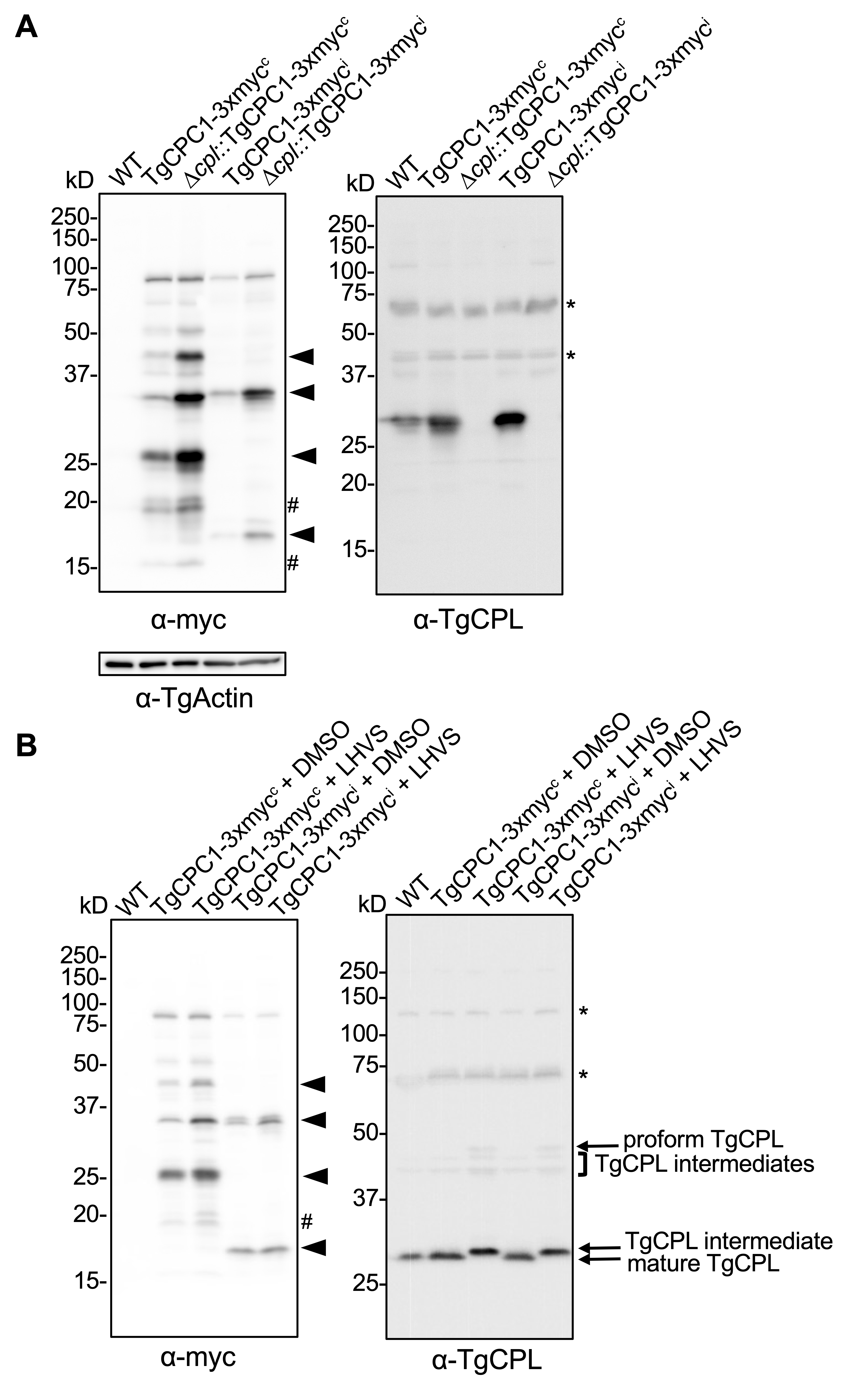

Supplement: Figure S2 — TgCPL is not involved in the intracellular cleavage of TgCPC1 but affects the abundance of TgCPC1. [file mbio.00174-23-s0002.tif]

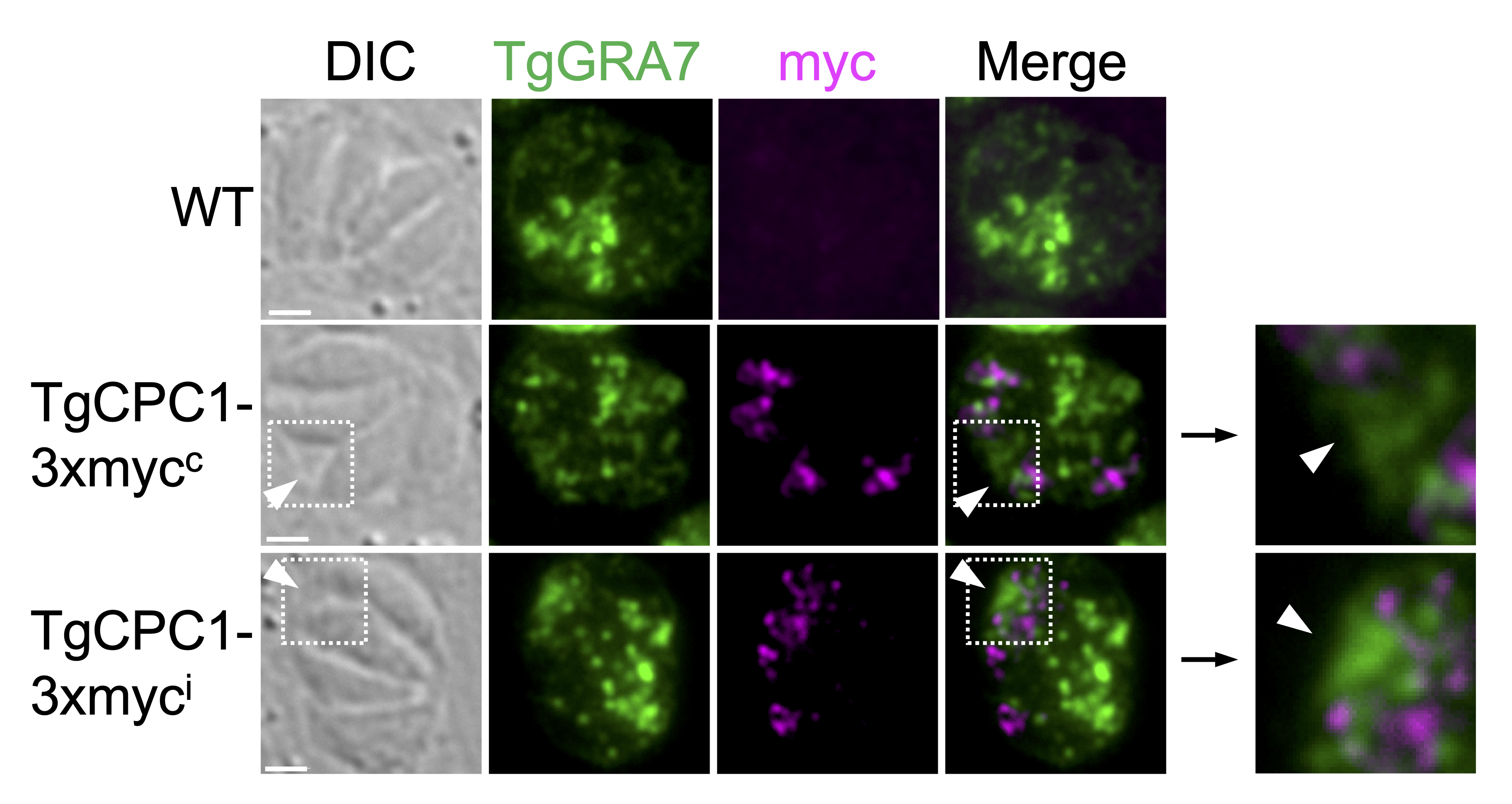

Supplement: Figure S3 — TgCPC1 is not detected in the PV. [file mbio.00174-23-s0003.tif]

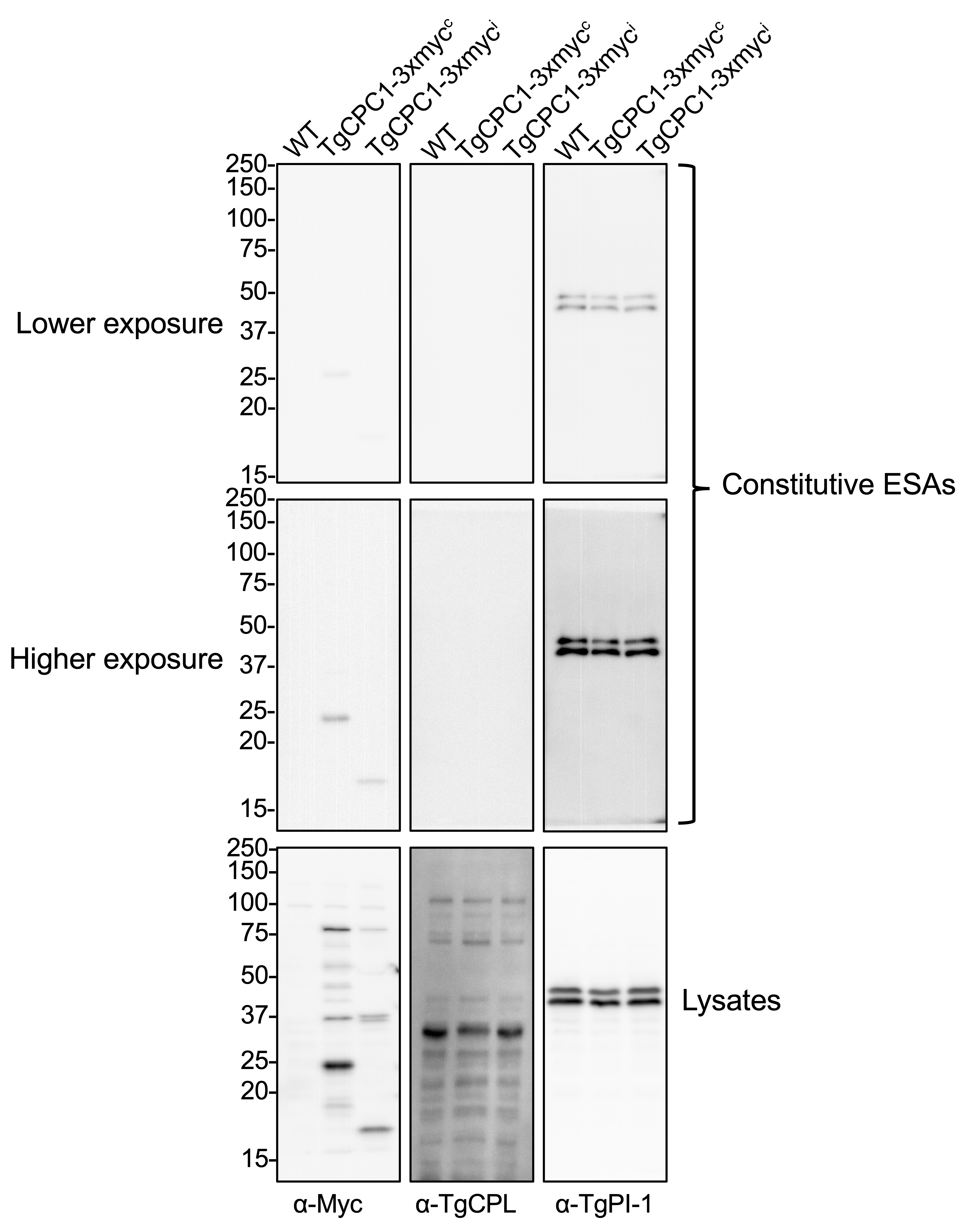

Supplement: Figure S4 — A trace amount of TgCPC1 is secreted by Toxoplasma parasites. [file mbio.00174-23-s0004.tif]

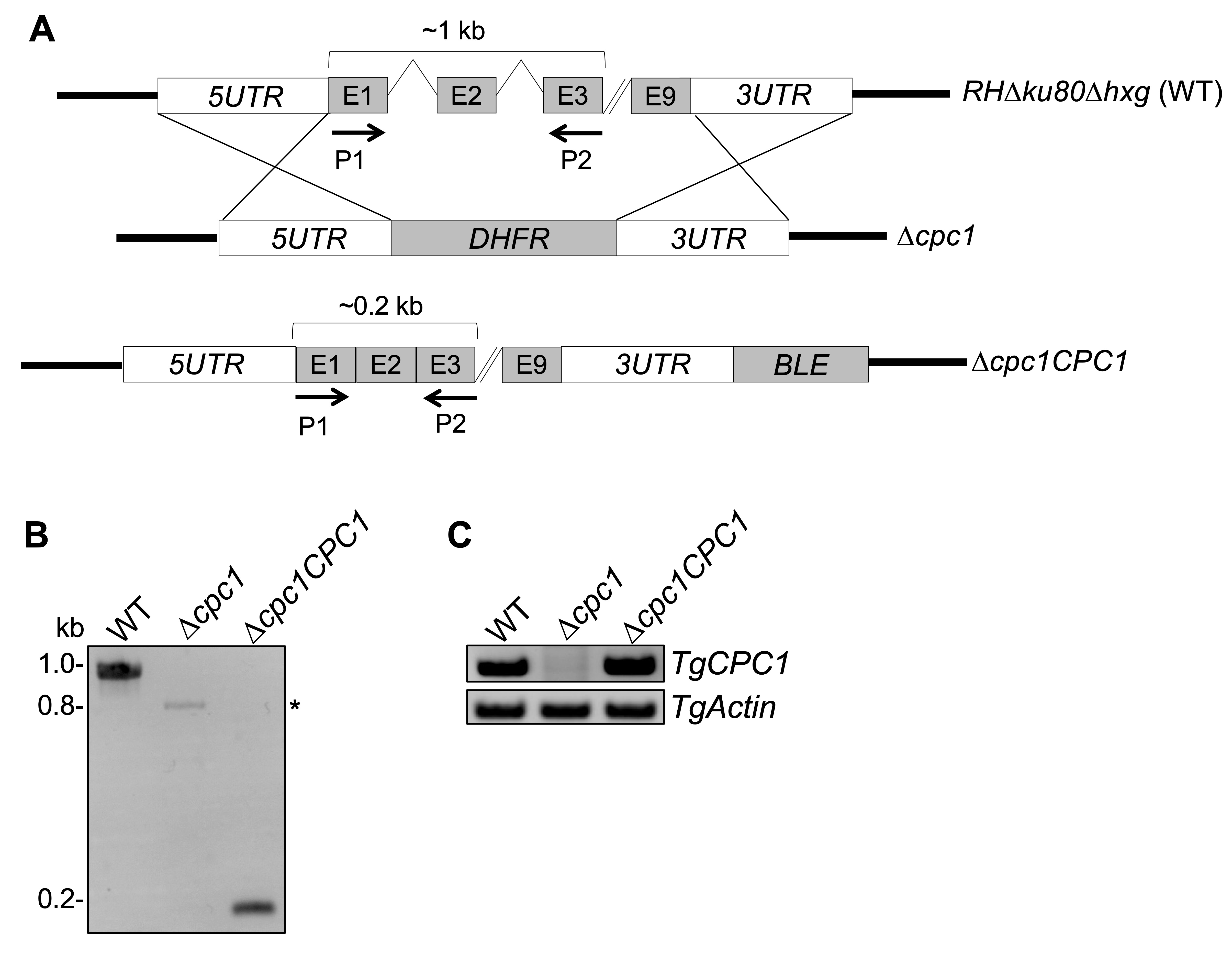

Supplement: Figure S5 — Generation of ∆cpc1 and ∆cpc1CPC1 strains. [file mbio.00174-23-s0005.tif]

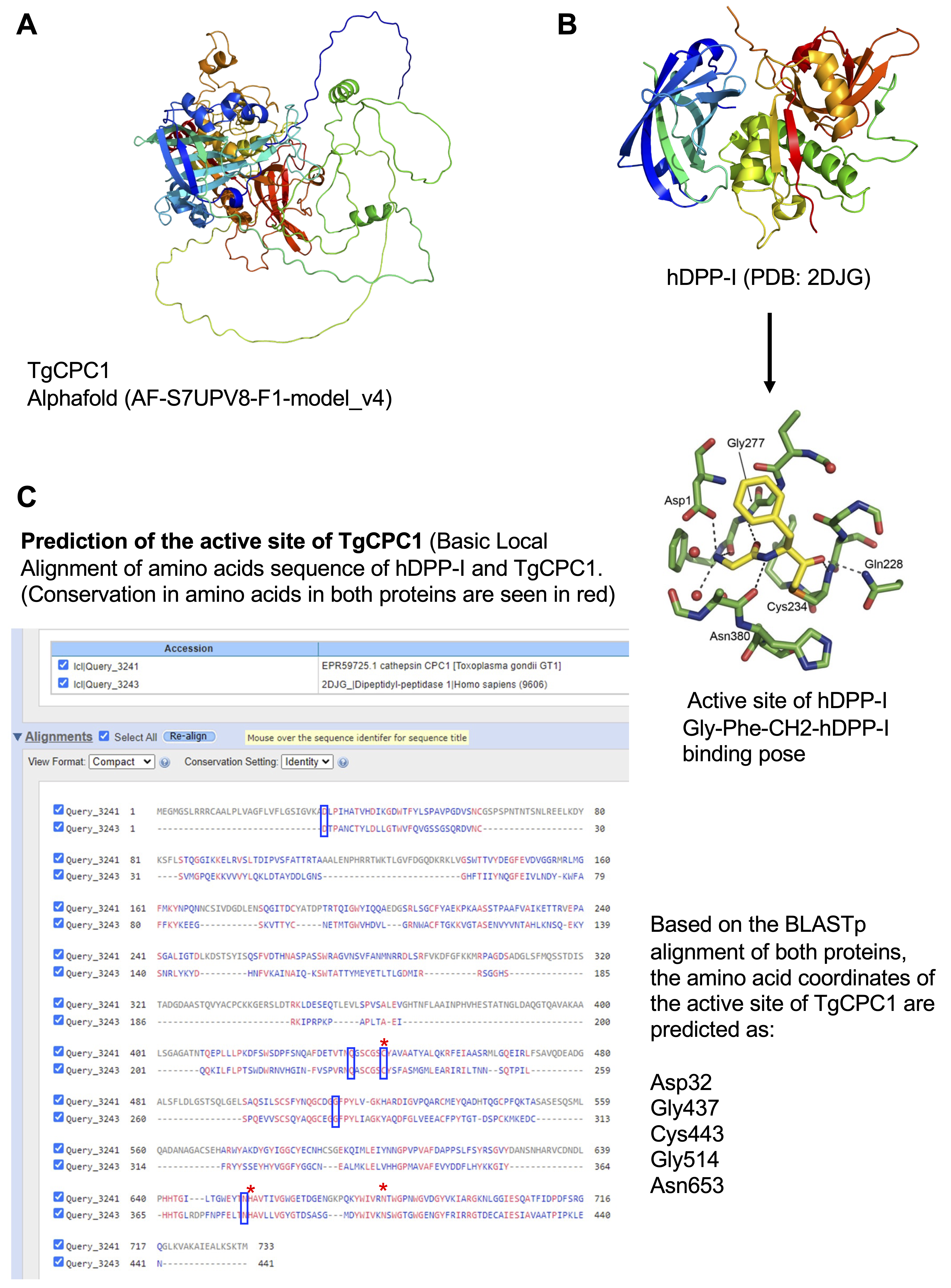

Supplement: Figure S6 — The prediction of the active sites of TgCPC1 interacting with BI-2051. [file mbio.00174-23-s0006.tif]

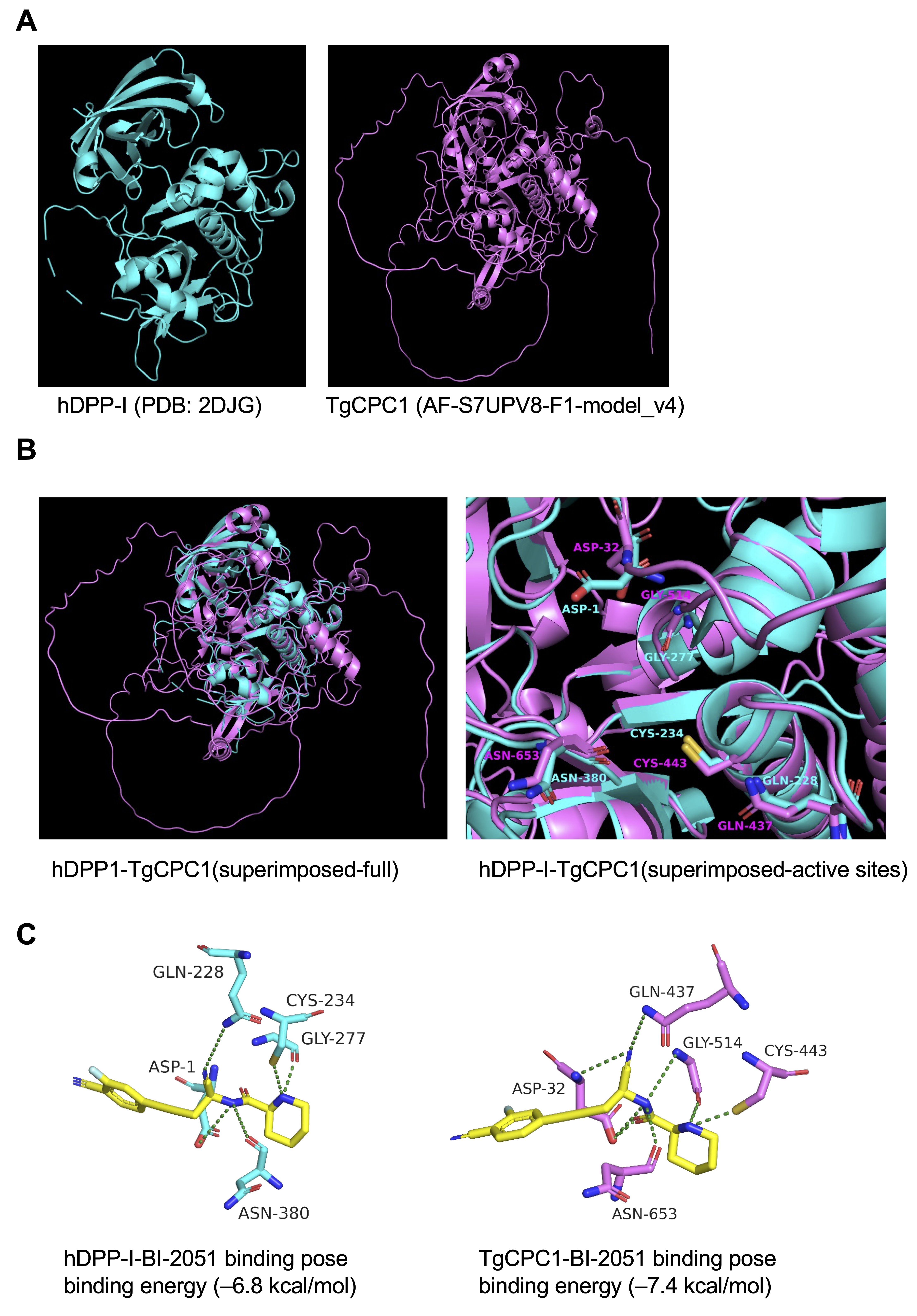

Supplement: Figure S7 — Molecular modeling of cathepsin C proteases with BI-2051. [file mbio.00174-23-s0007.tif]
