# Supplementary material for: A cathepsin C-like protease mediates the post-translation modification of Toxoplasma gondii secretory proteins for optimal invasion and egress
Source: mBio. 2023 Jun 16;14(4):e00174-23. doi: 10.1128/mbio.00174-23 (PMC10470614; doi:10.1128/mbio.00174-23)
Supplement: Supplemental Figure and Table Legends — Legends to Fig. S1 to S7 and Tables S1 to S3. [file mbio.00174-23-s0008.docx]

**Supplemental Figure and Table Legends**

**Figure S1. Primary structure and motifs of TgCPC1.** (A) TgCPC1 carries a putative signal peptide. The prediction of the signal peptide was performed using a SignalP 6.0 algorithm (https://services.healthtech.dtu.dk/service.php?SignalP-6.0). (B) Antigenic region prediction was conducted using EMBOSS program for the internal epitope-tagging of TgCPC1. The region within the red box was picked as the site for insertion of the 3xmyc epitope tag. (C) Primary structure and motif annotation in TgCPC1-3xmyc^c^ and TgCPC1-3xmyc^i^ strains. The cleavage sites denoted by solid black arrowheads were deduced by comparing against cleavage sites within human CPC protease and the homologous alignment between TgCPC1 and human DPP-I. The cutting site between the putative light and heavy chains, indicated by the hollow black arrowheads, was predicted from the observed molecular weights of cleaved TgCPC1 species shown in Fig. 1B. The essential Cys, His, and Asn within the catalytic triad, are labeled in red. Asterisks represent the stop site of translation.

**Figure S2. TgCPL is not involved in the intracellular cleavage of TgCPC1 but affects the abundance of TgCPC1.** (A) TgCPC1 was tagged with C-terminal and internal 3xmyc tags in ∆*cpl*. WT, TgCPC1-3xmyc^c^, ∆*cpl*::*TgCPC1-3xmyc^c^*, TgCPC1-3xmyc^i^, and ∆*cpl::TgCPC1-3xmyc^i^* parasites were grown in HFFs for 48 hrs before lysate preparation. Lysates were probed with anti-myc antibody to assess the cleavage patterns of TgCPC1. There were no distinguishable changes in TgCPC1 cleavage between WT and ∆*cpl* background, suggesting that TgCPL is not required for TgCPC1 proteolytic cleavage. TgCPL was also probed to confirm its loss in *TgCPL*-deletion strains. TgActin was included as a loading control. (B) To validate the observation shown in Fig. S2A, WT, TgCPC1-3xmyc^c^ and TgCPC1-3xmyc^i^ parasites were treated with 1 µM LHVS or DMSO (vehicle control) for 48 hrs before lysate preparation. Similar phenotypes were observed. The bands denoted with the arrowheads or the number signs in the blots probed by anti-myc antibody represented intermediates or degradation products of TgCPC1, respectively. The bands marked with the asterisks in the blots probed by anti-TgCPL antibody were non-specific staining.

**Figure S3. TgCPC1 is not detected in the PV.** To test if TgCPC1 is secreted into the PV, the replicated TgCPC1-3xmyc^c^ and TgCPC1-3xmyc^i^ parasites were co-immunostained with anti-TgGRA7 and anti-myc antibodies. The myc staining was contained within the parasites and was not observed in the PV space denoted by arrowheads. Bar = 2 µm.

**Figure S4. A trace amount of TgCPC1 is secreted by *Toxoplasma* parasites.** Purified extracellular WT, TgCPC1-3xmyc^c^ and TgCPC1-3xmyc^i^ parasites were subjected to the preparation of constitutive ESAs. The ESAs were probed with anti-myc, anti-TgCPL (negative control), and anti-TgPI-1 (positive control) antibodies. In contrast to TgCPL staining, a trace amount of TgCPC1 was observed in the ESA fractions, suggesting that TgCPC1 can be released from the parasites by an undefined pathway. At least two independent preparations of ESAs and total protein lysates were generated for this assay.

**Figure S5. Generation of ∆*cpc1* and ∆*cpc1CPC1* strains.** (A) Schematic representation of the approach used for generating ∆*cpc1* and for complementing the parasites with *TgCPC1*. WT parasites were transfected with a deletion construct containing a DHFR resistance cassette flanked by the 5’ and 3’ UTR regions that are upstream and downstream of the *TgCPC1* gene. Homologous recombination allowed for the replacement of the *TgCPC1* gene with the DHFR resistance cassette in order to generate ∆*cpc1*. The ∆*cpc1* parasites were complemented by introducing a plasmid containing the coding sequence of *TgCPC1* flanked by its own 5’ and 3’ UTRs in addition to a bleomycin (*BLE*) resistance cassette*.* (B) PCR verification of ∆*cpc1* and ∆*cpc1CPC1* strains. The PCR primers indicated in the schematic were used to verify the absence and complementation of the *TgCPC1* coding sequence (CDS) within ∆*cpc1* and ∆*cpc1CPC1*, respectively. The sizes of the corresponding PCR products were indicated in the schematic*.* The band marked with asterisk was from non-specific PCR amplification. (C) Quantitative PCR confirmed the loss and recovery of *TgCPC1* transcripts in ∆*cpc1* and ∆*cpc1CPC1* parasites*. TgActin* was included as a loading control.

**Figure S6. The prediction of the active sites of TgCPC1 interacting with BI-2051.** The protein sequences of hDPP-I and TgCPC1 were acquired from www.uniprot.org. (A) The predicted 3-D structure of TgCPC1 by Alphafold algorithm. (B) Prediction of the amino acid residues within the active site of hDPP-I that interact with BI-2051. (C) A global BLASTp program was used for alignment of hDPP-I and TgCPC1 to predict the amino acids within the active site of TgCPC1 that bind to BI-2051. The amino acids in red are the conserved residues. The essential amino acids within the catalytic triad, are marked with red asterisks. The predicted amino acid residues interacting with BI-2051 are boxed in blue rectangles.

**Figure S7. Molecular modeling of cathepsin C proteases with BI-2051.** (A) Crystal structure of hDPP-I and predicted structure of TgCPC1 by Alphafold algorithm. The three-dimensional structure of hDPP-I was acquired from the RCSB Protein Data Bank (PDB). The primary citation of related structures is 2DJG. (B) Superimposition of the entire structures and active sites of hDPP-I and TgCPC1 by Autodock Vina. (C) Spatial arrangement of the key residues within the active sites of hDPP-I and TgCPC1 that bind to BI-2051. The binding energy was calculated from the predicted conformation of protease-inhibitor interaction and reported in kcal/mol.

**Table S1. Primers used in the study.**

**Table S2. Parasite strains used in the study.**

**Table S3. Protein acronyms used in the paper.**
